# Safety and Feasibility of Vaginal Delivery in Full-Term Pregnancy After Transvaginal-Natural Orifice Transluminal Endoscopic Surgery: A Case Series

**DOI:** 10.3389/fsurg.2022.888281

**Published:** 2022-04-28

**Authors:** Shoufeng Zhang, Zhiyong Dong, Junling Liu, Zhenyue Qin, Huihui Wang, Mingyue Bao, Weiwei Wei, Ruxia Shi, Jiming Chen, Bairong Xia

**Affiliations:** ^1^ Dalian Medical University, Dalian, China; ^2^ Department of Obstetrics and Gynecology, the Affiliated Changzhou No. 2 People’s Hospital of Nanjing Medical University, Changzhou, China; ^3^ Department of Gynecology, The First Affiliated Hospital of USTC, Division of Life Sciences and Medicine, University of Science and Technology of China, Hefei, China

**Keywords:** transvaginal-natural orifice transluminal endoscopic surgery, Minimally invasive gynecology techniques, transvaginal delivery, full-term pregnancy, Childbirth ability

## Abstract

**Study Objective:**

The aim was to investigate the outcome of vaginal delivery of full-term pregnancies in patients after transvaginal-natural orifice transluminal endoscopic surgery (vNOTES) treatment for gynecological disorders.

**Design:**

A case series report.

**Setting:**

A medical university hospital.

**Patients:**

12 cases of successful delivery after transvaginal-natural orifice transluminal endoscopic surgery.

**Interventions:**

Long-term follow-up of patients with fertility needs after transvaginal-natural orifice transluminal endoscopic surgery.

**Measurements and Main Results:**

From 2018 to 2021, 163 cases of gynecological diseases were treated by vNOTES. One hundred forty-seven patients were followed up, with a follow-up rate of 90.1%. The average follow-up time was 28 (15–47) months, including 66 cases with fertility requirements. Among these 66 patients, 12 patients successfully got pregnant and completed delivery, including 10 cases of vaginal delivery and 2 cases of cesarean section, with no adverse pregnancy outcomes associated with vNOTES arising.

**Conclusion:**

Vaginal delivery of a full-term pregnancy after transvaginal-natural orifice transluminal endoscopic surgery appears to be safe and feasible and would not be one of the bases for elective cesarean delivery.

## Introduction

vNOTES is an emerging minimally invasive technique that enables surgical access to the peritoneal cavity through the vagina, a natural body orifice. In recent years, with the rapid development of minimally invasive gynecology and the concept of accelerated recovery surgery, combined with the unique advantages of scarless skin and fast recovery, vNOTES has made not only a splash in the field of gynecology (1) but also became an emerging surgical modality in general surgery (2, 3) and urology (4, 5).

Although the therapeutic efficacy and safety of vNOTES in the treatment of a variety of benign and malignant gynecological diseases have been demonstrated (6–8), there is still a lack of research on its long-term postoperative effects, such as the safety of vaginal delivery in full-term pregnancies and the impact on sexual life. In this study, we investigated the impact of vNOTES on vaginal delivery of full-term pregnancies after surgery in patients by retrospectively analyzing a case series.

## Materials and Methods

### Patients

This study retrospectively collected 163 patients with gynecological diseases treated by vNOTES in the Affiliated Changzhou No. 2 People’s Hospital of Nanjing Medical University from 2018 to 2021. 147 patients were followed up, with a follow-up rate of 90.1%. The average follow-up time was 28 (15–47) months. Among the 66 patients with fertility requirements, 12 cases were successfully pregnant and completed delivery, including 10 cases of vaginal delivery and 2 cases of cesarean section. See Supplementary Appendix S1 and Appendix S2 for case data.

### Surgical Technique

**Figure 1:**
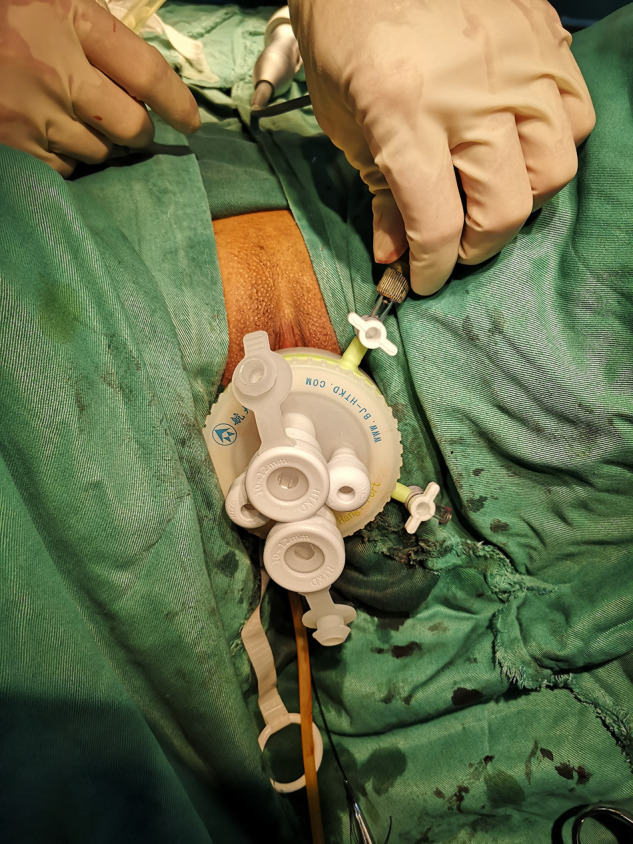
Establish vaginal access.

**Figure 2:**
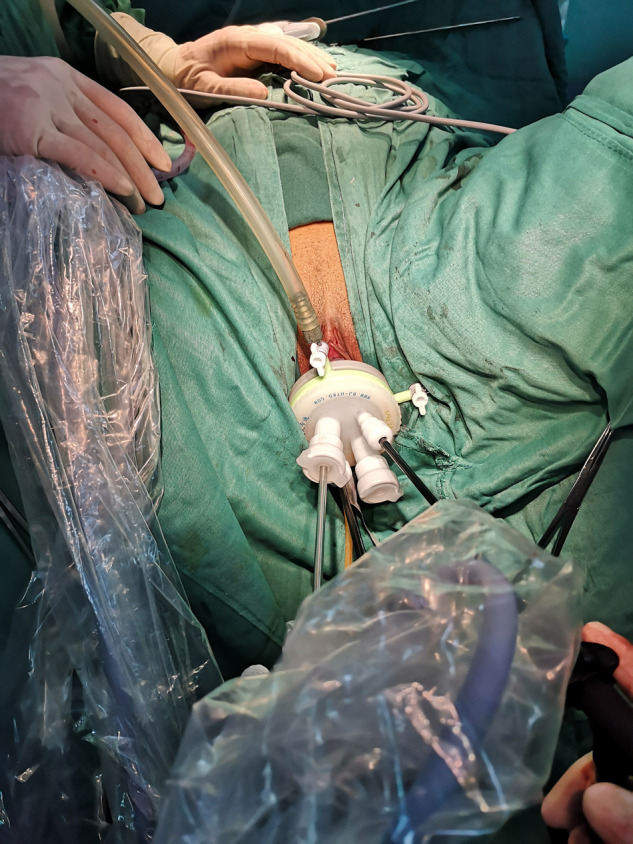
Schematic diagram of laparoscopic operation devices.

The patient requires vaginal cleansing one day before the procedure. Anterior vaginal vault approach: cervical forceps or Allis forceps the anterior cervical lip and pull downward, make a transverse incision slightly below the cervical portion of the bladder attachment, bluntly separate the vesicovaginal space, free the bulging bladder, separate the cervical ligament of the bladder and push the bladder upward to the retroperitoneum of the bladder and open the retroperitoneum of the bladder and uterus into the pelvis; posterior vaginal vault approach: cervical forceps or Allis forceps the rear cervical lip and pull upward to expose the posterior vaginal vault. A transverse incision of approximately 2–3 cm is made 1.5–2.0 cm below the cervix to separate the rectal space and enter the pelvis bluntly. The pneumoperitoneum was established by inserting a particular HangT Port (Beijing HangTian KaDi Technology R&D Institute, Beijing, China) vaginal access (**Figure 1**). A standard 10-mm rigid 30° laparoscope was used through 1 trocar, whereas 2 endoscopic instruments were used through the other two trocars (**Figure 2**); through the surgical platform, access to remove the lesion peritoneal and vaginal vault incisions were closed with a running Vicryl 2 suture. All procedures were performed by a chief surgeon with extensive experience in vNOTES surgery.

### Outcomes

Baseline characteristics of patients included age, body mass index (BMI), obstetric history, and history of previous pelvic surgery. Surgical correlates included time of surgery, location of the surgical incision, surgical approach, postoperative pathological diagnosis, need for conversion to laparoscopy or cesarean, and surgical complications as indicated by Clavien-Dindo classification. All patients were followed up at one week, 1, 3, and 6 months postoperatively and annually postoperatively. Assessment of the healing of the surgical incision, non-healing or delayed healing of the incision, abnormal sensation of the incision (pain, itching), rupture or fluid flow from the incision, narrowing or shortening of the vagina, and adhesion of the vault are noted as poor healing of the incision. Detailed obstetric and delivery data were recorded for all patients, such as the gestational week of delivery, pregnancy and delivery complications, the time interval from the first day after vNOTES to the next delivery, perineal incision rate, and grading of perineal rupture. After delivery of the placenta and three days after delivery, the vaginal vault is exposed using a speculum or vaginal puller to check for tears or bleeding from the surgical scar. All data were tallied by one physician and examined by two others.

### Statistical Methodology

Percentages, mean, and standard deviation were performed. Statistical analysis was carried out with IBM-Microsoft SPSS version 26.0.

## Results

### Patient Characteristics

During the study period, a total of 12 patients completed their pregnancies and delivered successfully, and the characteristics of all patients are shown in **Table 1**.

**Table 1:** Characteristics of 12 patients who delivered after vNOTES.

Characteristics	Total (*n* = 12)
Mean age ± SD, y	30.16 ± 6.22
Preoperative BMI ± SD, kg/m²	20.71 ± 3.74
Fertility history, *n* (%)	
*n* = 0	4 (33)
*n* = 1	8 (67)
*n* ≥ 2	0
Previous surgical history, *n* (%)	
Artificial abortion	2 (16)
Laparoscopic myomectomy	1 (8)
Cesarean	0
Others	0

*SD, standard deviation; BMI, body mass index.*

### Surgical Outcome

In this study, all 12 vNOTES patients had successful surgical completion. These included mature ovarian teratoma (*n* = 6), tubal ectopic pregnancy (*n* = 5), an ovarian cyst (*n* = 1); the mean operative time was 128.4 min and the longest operative time was 190 min; the operative incisions included anterior vaginal fornix incision (*n* = 6), posterior vaginal fornix incision (*n* = 6), and no additional vaginal wall injury due to surgical manipulation was observed in all patients; according to Clavien-Dindo classification, there were two postoperative complications, one postoperative incisional infection, which healed well after incisional dressing change (Grade 1 complication); one postoperative fever due to abdominal infection (Grade 2 complication), which improved after antibiotic treatment. The surgical incision healing was reviewed at 1 week, 1 month, 3 months, and 6 months after surgery. One of the 12 patients had poor surgical incision healing due to infection. After cleaning and dressing change, the incision healed well within one week after the operation, and the patient delivered successfully through vagina in the follow-up (See **Table 2**).

**Table 2:** Surgical data and postoperative review results of 12 patients.

	Total (*n* = 12)
Diagnosis, *n* (%)	
Ovarian teratomas	6 (50)
Tubal ectopic pregnancy	5 (42)
Oophoritic cyst	1 (8)
Operation time ± SD, min	128.4 ± 37.7
Surgical incision location, *n* (%)	
Anterior vaginal fornix	6 (50)
Posterior vaginal fornix	6 (50)
Laparoscopy or laparotomy, *n*	0
Clavien-Dindo classification, *n*	
1	1
2	1
≥3	0
Incision healing, *n*	
Good healing	11
Poor healing	1

*Poor healing is defined as nonunion or delayed healing of the incision, abnormal sensation of the incision (pain, itching), incision rupture or fluid flow, vaginal stenosis or shortening, and dome adhesion.*

### Pregnancy and Delivery Outcomes

Among the 66 patients with reproductive needs, 46(69.7%) cases were successfully pregnant, but 6 (13%) cases had an abortion, and 3(50%) cases were successfully pregnant after abortion. At present, there are 31(67.4%) patients during pregnancy. Twelve cases of pregnancy and delivery were successful, conception modes were classified as natural (*n* = 11), and assisted reproduction (*n* = 1), and all patients were examined during pregnancy according to the maternity program. Complications of pregnancy included gestational diabetes (*n* = 2), gestational obesity (*n* = 1), gestational hypothyroidism (*n* = 1), gestational mild anemia (*n* = 1), cord encirclement (*n* = 3), and premature rupture of membranes (*n* = 2). The 12 cases of successful delivery were vaginal delivery (*n* = 10) and cesarean delivery (*n* = 2). One cesarean delivery was due to a twin pregnancy, and the fetal position did not allow for vaginal delivery. The other was due to a previous history of obstructed labor. The patient refused the attempt of vaginal delivery. Ten vaginal deliveries were full-term pregnancies, singleton in the first position, normal deliveries (*n* = 8), obstructed deliveries (*n* = 2), and one obstructed delivery due to a previous history of obstructed labor. The other case was cervical edema during pregnancy, which was not significantly associated with the vNOTES procedure. No bleeding or tearing of the vNOTES surgical scar was detected after delivery of the placenta and on the third postpartum day. Six patients with perineal rupture were all with first-degree perineal rupture, which was not significantly associated with the vNOTES procedure. No bleeding or tearing of the vNOTES surgical scar was detected after delivery of the placenta and on the third postpartum day. The mean interval from the first postoperative day to the next delivery was approximately 21.8 months, with the shortest being 11.1 months. (See **Table 3**)

**Table 3:** Obstetric delivery outcomes of 10 patients undergoing vaginal delivery.

	Total (*n* = 10)
Mean gestational age of delivery, wks	39^+6^
Interval time ± SD, mos	21.89 ± 2.5
Induced labor mode, *n* (%)	
Natural induction of labor	6 (60)
Assisted induction of labor	4 (40)
Oxytocin induced labor	4
Balloon induced labor	2
Mode of delivery, *n* (%)	
Natural childbirth	8 (80)
Vacuum assisted	2 (20)
Obstetric forceps assisted	0
Anesthesia mode, *n* (%)	
None	7 (70)
Epidural anesthesia	3 (30)
Labor time ± SD, (h)	4.58 ± 0.62
First stage of labor	3.94 ± 0.38
Second stage of labor	0.52 ± 0.24
Third stage of labor	0.10 ± 0.008
Intrapartum hemorrhage ± SD, ml	175.0 ± 8.33
Neonatus	
Biparietal diameter ± SD, cm	9.31 ± 0.1
Head circumference ± SD, cm	33.04 ± 0.52
Abdominal circumference ± SD, cm	34.28 ± 0.51
Weight ± SD, g	3497 ± 118.3
Perineum, *n* (%)	
Complete perineum	2 (20)
Episiotomy	2 (20)
Perineal tear	6 (60)
Spontaneous labor, *n*	8
Dystocia, *n*	2

*wks, weeks; mos, months; h, hour; m, milliliter; g, gram.*

## Discussion

vNOTES is a minimally invasive surgical technique for treating disease after endoscopic access to the pelvic and abdominal cavity via the vagina, a natural cavity. It is the most used and developed surgical technique for trans-natural cavity surgery. vNOTES was widely used to treat benign gynecological diseases after Lee reported using vNOTES for tubal resection for tubal pregnancy in 2012 (9). In vNOTES, the intraoperative blood transfusion and hospital days are comparable to trans umbilical single-port laparoscopic surgery. Still, vNOTES has more advantages in postoperative pain relief, reduction of incisional fat liquefaction, and cosmetic results (6–8). In recent years, vNOTES has been gradually explored in gynecologic malignancies (10). Due to the significant advantage of no scar on the abdominal wall, vNOTES has been favored by many young women of reproductive age. In China’s open third-child policy, promoting fertility and reducing the cesarean section rate has been favored become a priority (11, 12). Therefore, we are concerned about the possible long-term effects of vNOTES on vaginal delivery in term pregnancies. It has been reported that full-term delivery can lead to rupture of the vaginal vault (13), but whether vNOTES will receive long-term benefits on vaginal delivery of full-term pregnancy and female sexual function has been less reported (14, 15).

The location of the incision depends mainly on the location of the lesion. Although the vNOTES approach of the posterior vaginal fornix is enough to complete the surgical treatment of most diseases in general surgery, urology, and gynecological surgery, due to the natural barrier of the uterus, the posterior vaginal fornix approach is still a difficult challenge for the lesions of the anterior wall of the uterus and the front of the pelvic cavity, and the incision of the anterior vaginal fornix can well solve this difficulty. For examples, anterior wall myomas and cesarean scar pregnancies are suitable for the anterior vaginal vault approach. In contrast, most adnexal diseases, posterior uterine wall myomas (16), and pelvic lymph node dissection (17) are more suitable for posterior vaginal vault incisions. Simultaneously, the posterior vault is more extensible, and the surgical specimen is easier to obtain intact than the anterior vault. In our study, we found that in many patients, especially those with endometriosis, the incidence of posterior pelvic adhesions is higher than that in the front of the pelvic cavity. Posterior vaginal fornix adhesions or the closure of the uterine rectum depression often led to the failure of the establishment of posterior vaginal fornix approach in vNOTES surgery and the conversion to laparoscopic surgery or rectal injury. In this study, ten vaginal deliveries included anterior vaginal vault incisions (*n* = 5) and posterior vaginal vault incisions (*n* = 5), and none of them had surgical scar tears during delivery. The current research data show that the anterior vaginal fornix incision and the posterior vaginal fornix incision have no relevant impact on the vaginal delivery of full-term pregnancy. With further follow-up, we will obtain more data to confirm this view. Vaginal preparation 1 day before surgery can effectively reduce the number of bacteria in the vagina and reduce the risk of intraoperative infection (18), and surgical incision healing is unlikely to result in abnormal incision sensation (pain, itching), incision rupture, or fluid flow, or vault adhesions that could affect the patient’s sexual life and ability to give birth. In addition, the surgical operation may damage the vaginal wall, or the suture may cause vaginal narrowing or shortening, which may affect the patient’s sexual function after surgery and thus reduce the probability of natural conception. At present, there are few research reports in this field (14).

The vNOTES produce an old surgical scar between the anterior and posterior vault of the cervix, which lacks extensibility relative to healthy tissue and may become a factor that delays the progress of labor or causes scar tearing during vaginal delivery—becoming an indication for the choice of cesarean delivery? In this study, 10 patients delivered vaginally were full-term pregnancies, and 2 were delivered by cesarean section, with a mean cesarean section rate of 16.6%, which is lower than the cesarean section rate of 39.2% in the region; the mean neonatal weight was 3,497 g, and the maximum neonatal weight was 4,220 g. No slow progression of labor or tearing of the scar was observed during delivery, which may suggest that vNOTES surgery does not full-term affect the way full-term pregnancy is delivered vaginally. The shortest postoperative interval between the patient’s surgery and full-term delivery was 11.1 months, with a mean time of 21.8 months. It may still be a topic for discussion about how long it takes after vNOTES to qualify for transvaginal delivery.

Younger patient age is a distinctive feature of the vNOTES surgery population with high estrogen production and estrogen’s ability to increase collagen deposition, increase wound strength, and promote healing of the vaginal vault surgical incision; low estrogen may increase inflammation production, prolong healing time, and affect wound healing relative to older age groups (19–21), and for those older than 45 years of age advanced maternal age, the safety of vaginal delivery after vNOTES for full-term pregnancies was not explored.

### Study Limitations

The retrospective case report of the small sample is an essential limitation of this study and there was no systematic sexual function assessment for all patients with fertility requirements after vNOTES.

## Conclusion

In this retrospective case series, all women who successfully conceived and delivered did not have adverse birth outcomes significantly associated with vNOTES; based on our data, vNOTES appears to be safe and feasible for vaginal delivery after a full-term pregnancy, does not become a basis for elective cesarean delivery, and has important implications for the promotion of vNOTES. Multicenter, randomized controlled studies are needed to confirm the long-term benefits of vNOTES for vaginal delivery and sexuality.

## Data Availability

The original contributions presented in the study are included in the article/Supplementary Material, further inquiries can be directed to the corresponding author/s.
