# Supplementary material for: Safety and Feasibility of Vaginal Delivery in Full-Term Pregnancy After Transvaginal-Natural Orifice Transluminal Endoscopic Surgery: A Case Series
Source: Front Surg. 2022 Apr 28;9:888281. doi: 10.3389/fsurg.2022.888281 (PMC9096787; doi:10.3389/fsurg.2022.888281)
Supplement: Supplementary file 1 [file Tables1.docx]

Appendix 1: Characteristics of 12 successful delivery cases

| Case | Patient age  (y) | BMI（kg/m^2^） | Obstetrical History | LESS  procedure | Pathologic | Operative Duration（min） | incision location（anterior/posterior vaginal fornix） | poor healing of the incision | interval time（months） |
| --- | --- | --- | --- | --- | --- | --- | --- | --- | --- |
| 1 | 31 | 23.76 | G2P1 | Excision of left ovarian teratoma | findings | 190 | posterior | none | 12.8 |
| 2 | 34 | 26.48 | G3P1 | Left  cystectomy | Mature cystic | 110 | posterior | none | 11.1 |
| 3 | 25 | 24.41 | G1P0 | Left  cystectomy | Teratoma | 146 | anterior | none | 16.8 |
| 4 | 27 | 22.68 | G1P0 | Right  cystectomy | Mature cystic | 170 | anterior | none | 17.4 |
| 5 | 28 | 23.03 | G1P0 | Right salpingectomy | teratoma | 120 | posterior | none | 17.9 |
| 6 | 34 | 34.79 | G3P1 | Right salpingectomy | Mature cystic | 110 | posterior | none | 22.2 |
| 7 | 26 | 26.51 | G2P1 | Right  cystectomy | teratoma | 75 | anterior | none | 27.8 |
| 8 | 39 | 26.16 | G3P1 | Left  cystectomy | Mucinous | 115 | anterior | YES | 33.0 |
| 9 | 45 | 25.47 | G3P1 | Left  cystectomy | cystadenoma | 85 | posterior | none | 27.2 |
| 10 | 31 | 34.95 | G2P1 | Right salpingectomy | fallopian tube ectopic pregnancy | 165 | anterior | none | 32.7 |
| 11 | 26 | 24.84 | G1P0 | Right salpingectomy | fallopian tube ectopic pregnancy | 90 | anterior | none | 21.0 |
| 12 | 38 | 26.49 | G3P1 | Right  cystectomy | Mature cystic | 165 | posterior | none | 44.5 |

Note: The interval time refers to the time from the first day after operation to the next delivery. The 11th and 12th cases were cesarean sections, and the rest were vaginal delivery.

Appendix 2: Vaginal delivery outcomes of 10 cases

| CASE | The gestational age at delivery(wks) | Biparietal diameter（cm） | Head circumference (cm) | Abdominal circumference (cm) | Neonatal weight（g） | Apgar score（1min-5min-10min） | Induced labor mode | production process（the first of labor- the second of labor- the third of labor）（t） | Labor analgesia | Delivery complications |
| --- | --- | --- | --- | --- | --- | --- | --- | --- | --- | --- |
| 1 | 40^+2^ | 9.9 | 34.6 | 36.8 | 4220 | 9、10、10 | spontaneous parturition | 4.80（4.33-0.33-0.13） | Epidural anesthesia | First-degree rupture of the perineum |
| 2 | 39^+4^ | 9.0 | 31.2 | 32.3 | 3500 | 9、10、10 | spontaneous parturition | 3.92（3.67-0.17-0.08） | None | First-degree rupture of the perineum |
| 3 | 39^+5^ | 9.3 | 32.1 | 33.4 | 2950 | 9、10、10 | Oxytocin induces labor | 3.42（3.08-0.23-0.10） | Epidural anesthesia | First-degree rupture of the perineum |
| 4 | 40 | 9.4 | 33.7 | 35.2 | 3700 | 9、10、10 | Vacuum Extraction | 4.58（4.00-0.43-0.15） | Epidural anesthesia | III-degree amniotic fluid contamination +fetal head dystocia |
| 5 | 40^+5^ | 9.7 | 36.8 | 34.8 | 3600 | 9、10、10 | cervical expansion ballon+ artificial rupture of membranes+ Oxytocin induces labor | 9.83（7.00-2.75-0.08） | Epidural anesthesia | None |
| 6 | 39^+5^ | 9.5 | 32.9 | 33.6 | 3400 | 9、10、10 | spontaneous parturition | 3.33（3.00-0.20-0.13） | None | III-degree amniotic fluid contamination |
| 7 | 40 | 8.9 | 32 | 35.6 | 3800 | 9、10、10 | spontaneous parturition | 2.83（2.50-0.20-0.13） | None | First-degree rupture of the perineum |
| 8 | 40 | 8.9 | 31.4 | 31.8 | 3000 | 9、10、10 | Oxytocin induces labor | 3.83（3.50-0.25-0.08） | Epidural anesthesia | None |
| 9 | 39^+2^ | 9.4 | 32.7 | 33.5 | 3500 | 9、10、10 | spontaneous parturition | 4.50（4.08-0.33-0.08） | None | None |
| 10 | 39^+2^ | 9.1 | 33 | 35.8 | 3300 | 9、10、10 | cervical expansion ballon+ artificial rupture of membranes+ Vacuum Extraction | 4.83（4.33-0.37-0.13） | None | First-degree rupture of perineum + fetal head dystocia |
